# Supplementary material for: Fabrication of EP@PDMS@F-SiO2 Superhydrophobic Composite Coating on Titanium Alloy Substrate
Source: Biomimetics (Basel). 2025 Jun 16;10(6):404. doi: 10.3390/biomimetics10060404 (PMC12190485; doi:10.3390/biomimetics10060404)
Supplement: Supplementary file 1 [file biomimetics-10-00404-s001.zip › biomimetics-3646619-supplementary.pdf]

Ⅰ. Fig. S1. Surface morphology and wettability of fluorinated and unmodified silica particle coatings.

(a) represents the fluorinated silica ($F-SiO_2$) particle coating; (b) represents the unmodified silica (SiO_2) particle coating. In both cases, the water droplets exhibit well-defined contact angles, indicating hydrophobic and superhydrophobic surface characteristics. The significantly enhanced hydrophobicity observed in (a) can be attributed to the synergistic effect of micro/nanoscale hierarchical roughness and the low surface energy introduced by fluorination. The water droplet rests on the top of the surface asperities, forming a stable Cassie–Baxter state, which minimizes the solid–liquid contact area, reduces adhesion, and imparts excellent self-cleaning ability to the surface.

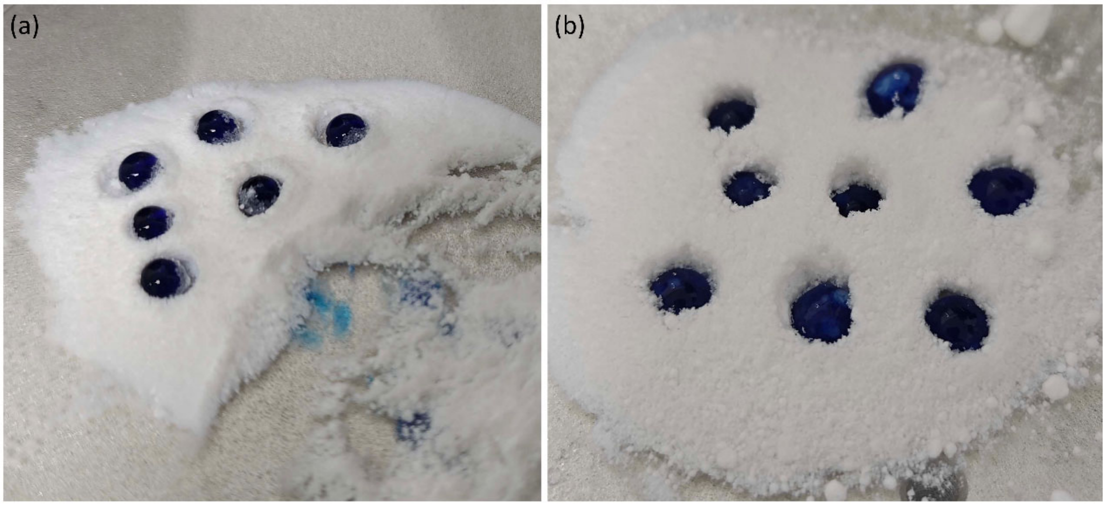

Fig. S1. Surface morphology and wettability of fluorinated and unmodified silica particle coatings. (a) Fluorinated silica ($F-SiO_2$) particle coating; (b) unmodified silica (SiO_2) particle coating.

Ⅱ. Fig .S2 Three-dimensional schematic of the coating surface of Sample 3.

Fig S2 illustrates a three-dimensional schematic of the coating surface of Sample 3, prepared using fluorosilane (FAS)-modified silicon dioxide (SiO_2) particles. As shown in the figure, the SiO_2 particles are uniformly distributed and densely packed within the coating, forming a hierarchical micro/nano-scale rough structure. These particles exhibit a clustered and textured morphology, which significantly reduces the contact area between water droplets and the solid surface. This structural configuration facilitates the formation of a classic Cassie–Baxter wetting regime, thereby imparting excellent superhydrophobic properties to the coating.

In addition, no significant particle agglomeration is observed, indicating that FAS modification effectively improves the dispersion of the SiO_2 particles. This enhanced dispersion contributes to the uniformity and integrity of the surface structure, which not only enhances the hydrophobic performance but also greatly improves the mechanical stability and durability of the coating under friction and environmental stress.

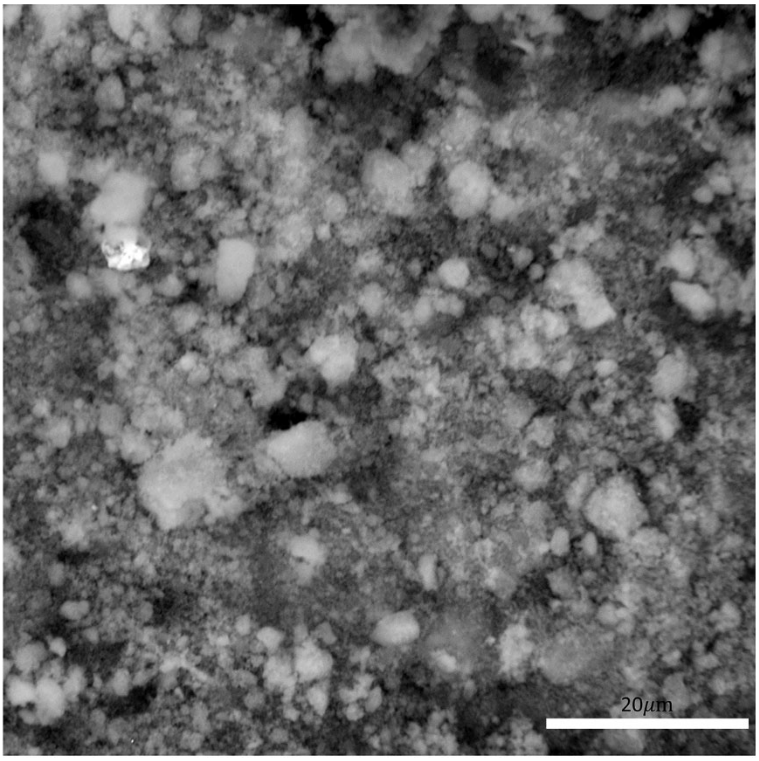

Fig.S2 Three-dimensional schematic of the coating surface of Sample 3.
